# Supplementary material for: Validation of survey effort measures of grit and self-control in a sample of high school students
Source: PLoS One. 2020 Jul 1;15(7):e0235396. doi: 10.1371/journal.pone.0235396 (PMC7329102; doi:10.1371/journal.pone.0235396)
Supplement: S1 Appendix — (DOCX) [file pone.0235396.s001.docx]

*APPENDIX*

Table A.1

*Description of Scales Included in the Student’s Careless-Answering Measure*

| **Scale** | **Reliability** | **Items** | **Average Absolute Residuals** |
| --- | --- | --- | --- |
| *Trust* | 0.71 | *I am treated fairly by teachers and other adults at my high school.* | 0.57 |
|  |  | *Teachers and other adults treat students in my racial group with respect.* | 0.62 |
|  |  | *Teachers give me the grades I think I deserve.* | 0.75 |
|  |  |  |  |
| Belonging | 0.79 | Sometimes I worry that I will not belong in college. | 0.60 |
|  |  | I am anxious that I will not fit in at college. | 0.60 |
|  |  | I feel confident that I will belong in college. | 0.73 |
|  |  | When I face difficulties in high school, I wonder if I will really fit in when I get to college. | 0.79 |
|  |  |  |  |
| Interest in School | 0.82 | I like school more than most of my other activities. | 0.81 |
|  |  | I find working on school assignments interesting. | 0.71 |
|  |  | I like schoolwork. | 0.69 |
|  |  |  |  |
| Academic Self-Efficacy | 0.86 | I know I can learn the material in my classes. | 0.38 |
|  |  | I believe that I can be successful in my classes. | 0.36 |
|  |  | I am confident that I can understand the material in my classes. | 0.47 |
|  |  |  |  |
| Distress Tolerace | 0.79 | Feeling distressed or upset is unbearable to me. | 0.73 |
|  |  | I can't handle feeling distressed or upset. | 0.73 |
|  |  | There's nothing worse than feeling distressed or upset. | 0.86 |
|  |  |  |  |
| Purpose | 0.72 | I want to learn things that will help me make a positive impact on the world. | 0.53 |
|  |  | I want to gain skills that I can use a job that helps others. | 0.57 |
|  |  | I want to become an educated citizen who can contribute to society. | 0.61 |
|  |  |  |  |
| Brief Self-Control | 0.73 | I have a hard time breaking bad habits. | 0.80 |
|  |  | I do certain things that are bad for me, if they are fun. | 0.79 |
|  |  | Pleasure and fun sometimes keep me from getting work done. | 0.79 |
|  |  | I have trouble concentrating. | 0.83 |
|  |  | Sometimes I can't stop myself from doing something, even if I know it's wrong. | 0.74 |
