# Supplementary material for: Validation of survey effort measures of grit and self-control in a sample of high school students
Source: PLoS One. 2020 Jul 1;15(7):e0235396. doi: 10.1371/journal.pone.0235396 (PMC7329102; doi:10.1371/journal.pone.0235396)
Supplement: Attachment — Submitted filename: PLOS November 2019.docx [file pone.0235396.s002.docx]

Manuscript Number: PONE-D-19-29452

Title: Further Validation of Survey Effort Measures of Relevant Character Skills: Results from

a Sample of High School Students

The manuscript presents evidence in the validation of survey effort measures in predicting survey and behavior assessments of grit and self-control. This study was ambitious in the sense that it collected several behavioral and teacher-report assessments of over 500 children. There are, however, some issues that need to be addressed. Below, I list several suggestions for improvement (though not necessarily in order of importance).

1. This manuscript includes several grammatical errors and would benefit substantially from thorough copy editing. Moreover, the organization of this manuscript (e.g., the subhead “Data” should be “Materials and Methods”) does not fit with APA guidelines and that of PLOS (<https://journals.plos.org/plosone/s/submission-guidelines>). Finally, as per APA guidelines, please report measures and results in past tense. These errors and issues limit the manuscripts readability quite a bit.
2. Use of the term “character skills” to describe the personality trait conscientiousness and its facets is odd. Decades of research has identified conscientiousness as a personality trait. Please include rationale for why conscientiousness and its facets are termed as a character skill and not a personality trait in this context. Alternatively, given that conscientiousness is not measured directly, I suggest focusing on grit and self-control throughout the manuscript, especially the methods and results sections.
3. Relatedly, you introduce the manuscript’s focus on validation by stating that “researchers have struggled to find valid measures of these skills with many existing datasets lacking any measures at all”. In fact, measures to assess trait level conscientiousness have been very well validated (see BFI, BFI2, NEO, etc.) If you decide to focus on conscientiousness and its facets, this sentence is problematic.
4. Please include reference to the measures you used as well as example items for each.
5. In the Survey Effort Measures section, please clarify what constitutes as “legitimate skips” with regards to item non-responding.
6. Please clarify why teachers rated the grit measure items as a composite instead of each item individually. This approach limits the measures’ predictive power as well as its ability to assess the measures’ reliability. Also, please report the number of students each teacher gave ratings for.
7. Clarify what including self-report measures “for completeness” means in the context of this study.
8. Please explain how and why the measures for grit and self-control were adapted from the original scales.
9. Its my understanding that GPA was on a scale from 1.0-4.0. Please explain why your measure of GPA is on a scale from 1 to 100.
10. Please report effect sizes of regression models throughout results sections instead of or in addition to standard deviation increases or decreases.
11. Please include a note for Table 1 explaining what ELL, SPED etc. mean.
12. In the discussion section, address limitations for the lack of variation in outcome measures (e.g., graduating high school). Relatedly, please address the limitations of your item non-response measure using (1) a continuous measure with 47% of participants’ completing all questions (2) a dichotomous measure of non-responding is problematic (i.e., not answering 1 question is not the same as not answering 10 questions), as well as the implications of the non-normal distribution for this measure (e.g., that careless responding may be a better predictor).
13. While you do recognize the small effect sizes (i.e., “Although the magnitudes of the correlations between survey effort and survey self-reported measures may appear small, they are at least as large as the correlations reported in prior literature validating other behavioral-task measures of conscientiousness, grit, and self-control.”), please address this limitation in the discussion section and caution readers in over-interpreting small effect sizes. Specifically, we may trust some effects more than others. Likewise, some effects may require replication more than others.
